# Comment from the Editors on the Special Issue: Advanced Analytical Methods in Clinical Diagnosis and Therapy

**DOI:** 10.3390/jcm8111936

**Published:** 2019-11-10

**Authors:** Monica Florescu, Liliana Rogozea

**Affiliations:** Faculty of Medicine, Transilvania University of Brasov, 500019 Brasov, Romania; r_liliana@unitbv.ro

**Keywords:** analytical methods, diagnosis, therapy, clinical laboratory, medical imaging, telemedicine, rehabilitation medicine

## Abstract

With this Editorial, we want to present the Special Issue, “Advanced Analytical Methods in Clinical Diagnosis and Therapy”. The development of medicine is not possible without progress in the field of identifying different biomarkers or treatments using modern approaches, such as the analytical methods presented in articles that are part of this issue. Thus, with the support of experts, both aspects of theoretical and practical interest from different fields of pathologies have been addressed.

The use of biomarkers is becoming more and more common for various purposes, e.g., detection, monitoring, and treatment of the molecular causes of diseases; molecular markers and genetic variations that affect drug response, development of new drugs, adverse effects and/or disease progression; new tools and technologies for diagnosis and therapy; and ethical and regulatory issues related to personalized medicine. 

This Special Issue publishes manuscripts (original research and review articles) dealing with advances in healthcare/clinical practices, studies of direct observation of patients, and general medical research works. Some of the manuscripts are presented in the framework of the International Conference on Analytical and Nanoanalytical Methods for Biomedical and Environmental Sciences, IC-ANMBES 2018 (https://sciforum.net/conference/icanmbes2018). 

A DNA methylation-based test for breast cancer detection in circulating cell-free DNA has been proven to enable breast cancer accurate diagnosis and prognostic stratification in tissue samples, and allows for early detection in liquid biopsies. A case-control study to evaluate whether genetic polymorphisms of HOX transcript antisense RNA (HOTAIR) are associated with urothelial cell carcinoma risk has been presented and the results verified the diverse impacts of HOTAIR variants on UCC susceptibility and clinicopathologic characteristics. A retrospective cohort study evaluated the association between traditional Chinese medicine for depression and mortality in patients with prostate cancer; the result show that TCM may have a positive association with the survival of prostate cancer patients with depression. A meta-analysis of randomized, controlled phase II or III trials to evaluate the clinical benefits of statins added to systemic anticancer therapy in patients with solid cancer has also been presented. The results do not support the clinical benefits of statins added to systemic anticancer therapy in patients with solid cancer.

Another study, which aimed to identify the association between CRP/albumin ratio and 28-day mortality and predict the accuracy of C-reactive protein/albumin ratio for 28-day mortality in medical intensive care unit patients, concludes that a higher CRP/albumin ratio is associated with increased mortality in critically ill patients. In addition, a retrospective cohort study found that hypoalbuminemia on emergency department arrival is a reliable predictor for in-hospital mortality in necrotizing fasciitis patients. 

Recent trends in the quantification of biogenic amines in biofluids as useful biomarkers for the diagnosis, therapy, and prognosis of several neuroendocrine and cardiovascular disorders, including neuroendocrine tumors, schizophrenia and Parkinson’s Disease, are reported and critically discussed. The possibility of miRNAs becoming potential biomarkers in endometriosis and ERONs is discussed in order to gain a better understanding of whether, how, and when miRNAs might be used as biomarkers for early detection and accurate diagnosis of endometriosis and endometriosis-related ovarian neoplasms (ERONs). 

The research herein has complied with the ethical regulations in force and concerns for good quality research, in line with international regulations [1].

Thus, these original research papers and reviews that describe the development, characterization/evaluation, and utilization of different advanced analytical methods in clinical diagnosis and therapy have provided an up-to-date and critical overview of the state of the art of these methods.
